# The mitochondrial signaling peptide MOTS-c improves myocardial performance during exercise training in rats

**DOI:** 10.1038/s41598-021-99568-3

**Published:** 2021-10-11

**Authors:** Jinghan Yuan, Manda Wang, Yanrong Pan, Min Liang, Yu Fu, Yimei Duan, Mi Tang, Ismail Laher, Shunchang Li

**Affiliations:** 1 Institute of Sport Medicine and Health, Chengdu Sport University, Chengdu, 610041 China; 2 Department of Pharmacology and Therapeutics, Faculty of Medicine, University of British Columbia, Vancouver, BC V6T 1Z3 Canada

**Keywords:** Cardiac hypertrophy, Cardiovascular diseases, Heart development

## Abstract

Cardiac remodeling is a physiological adaptation to aerobic exercise and which is characterized by increases in ventricular volume and the number of cardiomyocytes. The mitochondrial derived peptide MOTS-c functions as an important regulator in physical capacity and performance. Exercise elevates levels of endogenous MOTS-c in circulation and in myocardium, while MOTS-c can significantly enhance exercise capacity. However, the effects of aerobic exercise combined with MOTS-c on cardiac structure and function are unclear. We used pressure–volume conductance catheter technique to examine cardiac function in exercised rats with and without treatment with MOTS-c. Surprisingly, MOTS-c improved myocardial mechanical efficiency, enhanced cardiac systolic function, and had a tendency to improve the diastolic function. The findings suggest that using exercise supplements could be used to modulate the cardiovascular benefits of athletic training.

## Introduction

Regular exercise improves cardiac function and prevents and treats cardiovascular diseases(CVD)^1,2^. The cardiac benefits of exercise are related to increases in myocardial oxygen supply and consumption, reductions in myocardial fibrosis and cell apoptosis, promoting angiogenesis, improving myocardial cell survival and cardiac function in CVD^3^. Physiological myocardial hypertrophy occurs when long-term exercise increases cardiac remodeling^4,5^, which is a physiological adaptation to aerobic exercise caused by increases in ventricular volume and the number of cardiomyocytes^6,7^. Aerobic exercise regulates mitochondrial dynamics, autophagy, and biogenesis to regulate cardiac hypertrophy^8–10^.

The mitochondrial-derived peptide MOTS-c (mitochondrial open-reading frame of the twelve SrRNA type-c) is a peptide recently discovered by Lee et al., containing 16 amino acid, and it is expressed mainly in the blood plasma, skeletal muscle and heart, both in rodents and human being^11,12^. This peptide performs various function, as an important cyto-protector in helping to maintain the mitochondrial function and the cellular viability under stressful conditions, (such as exercise), which has led several groups^13–15^ to propose that MOTS-c could mimic the benefits of exercise training. The cardiovascular effects of MOTS-c have mostly focused on cardiovascular effects such as coronary endothelial dysfunction and pathological myocardial remodeling^16–19^. However, the combined effects of MOTS-c and aerobic exercise on physiological myocardial remodeling has not been reported.

We examined the effects of MOTS-c on cardiac function (using Millar carotid catheter monitoring) and structure (using hematoxylin–eosin (HE) staining, transmission electron microscopy, and echocardiography) in rats exposed to chronic aerobic exercise. In addition, we also explored whether MOTS-c enhanced physiological cardiac adaptations to exercise training. Our findings provide an experimental basis for the use of putative exercise supplements to modulate the cardiovascular benefits of athletic training.

## Materials and methods

### Experimental animals

Twenty-four male Sprague–Dawley rats (6 weeks old) were housed in the Institute of Sports Medicine and Health at Chengdu Sport University. The animals were housed 5 to a standard rodent cage, maintained on a 12-h light /12-h dark cycle, and allowed access to food and water ad libitum. The animal room temperature was 21 °C–23 °C, and relative humidity was 40–60%. The protocols were conducted in accordance with the Turkish Legislation for the Use and Care of Laboratory Animals and were approved by the Experimental Animal Ethics Committee of Chengdu Sport University. The study was carried out in compliance with the ARRIVE guidelines.

The rats were randomly divided into three equal sized groups (8 rats per group): control (C), exercise training (E), exercise training combined with MOTS-c treatment (ME).

### Intervention protocols

#### Exercise training

All rats in the E and ME groups were adaptively trained on a treadmill (DSPT-208, Duan Animal Treadmill Co. Ltd, Hangzhou, China) for 1 week. In the formal exercise intervention, the treadmill started at a speed of 10 m/min, and gradually increased to 20 m/min at an inclination of + 10°. The exercise training lasted for 60 min per session and occurred 5 days a week for 12 weeks.

#### MOTS-c treatment

Rats were inject with MOTS-c and saline half an hour before the start of the exercise protocol. Rats in the ME group were injected intraperitoneally (i.p.) with MOTS-c (0.5 mg/kg/day) for 12 weeks^12^, while those in the C and E groups were treated with saline (0.5 mg/kg/day intraperitoneally) for 12 weeks.

### Hematoxylin–Eosin staining(HE)

HE staining was used to observe changes in gross cardiac structure of the rats. Left ventricular myocardial tissue was fixed with 4% paraformaldehyde and embedded in paraffin and then cut into 4 μm thick serial sections. Sections were deparaffinized in a sectioning machine (LEICA RM2245) and then treated with xylene, stained with hematoxylin–eosin, and dehydrated using graded solutions of alcohol. Sections were preserved and photographed in a neutral gum mount.

### Transmission electron microscope (TEM)

The apex of left ventricular was fixed in 3% glutaraldehyde, rinsed with buffer solution twice, and then stored overnight at 4 °C. The sample was then fixed with 1% osmium tetroxide for another 2 h, and rinsed with buffer solution twice. Acetone (30%, 50%) was used for stepwise dehydration at 4 °C for 10 min, followed by 70% acetone at 4 °C for 15 min, 90% acetone for 15 min, and finally, anhydrous acetone at room temperature (once every 10 min, twice in total, for a total of 20 min). The dehydrated sample was passed through a dehydrating agent and an epoxy resin (Epon812) permeate successively, using ratios of 3:1, 1:1, and 1:3, with each step lasting 30 to 60 min. The infiltrated sample was placed in a suitable mold, filled with embedding liquid to form a solid matrix (embedded block) after heating and polymerization, and prepared for sectioning. Semi-thin Sects. (1 μm) were used to create ultra-thin sections (~ 50 nm thick) using an ultra-thin microtome. Cut sections floated on the liquid surface of the knife tank and placed on a copper net. The sections were first stained with uranium acetate for 10 ~ 15 min (at room temperature), and then with lead citrate for 1–2 min. Changes of myocardial ultrastructure were observed with a JEM-1400 Flash transmission electron microscope.

### Echocardiography

A small animal echocardiograph (Philips CX50, Netherlands) was used, with a 11.5 MHz frequency of the system probe (S12-4). Rats were anesthetized with 1% sodium pentobarbital (0.15 ml/100 g, i.p.) and the cardiac area were shaved. The M-mode ultrasound test through the parasternal left ventricular long axis of the rats was used to record heart rate (HR), end-diastolic left ventricular diameter (LVIDd), end-diastolic left ventricular volume (EDV), ejection fraction (EF), and fractional shortening (FS). The E/A ratio was determined by measuring the peak blood flow velocities during early diastole (E) and late diastole (A) as recorded by the blood flow doppler. All values were obtained from the average of 3 consecutive cardiac cycles, with all tests performed by the same experimenter using the same instrument.

### Hemodynamic measurements: P–V-loop analysis

Hemodynamic changes of left ventricular function were detected by carotid left ventricular intubation. Rats were injected with 1% pentobarbital sodium (0.15 ml/100 g, i.p.), tracheotomized and intubated to facilitate breathing. Animals were placed on controlled heating pads, and their core temperature maintained at 37 °C. A cut (5–7 cm in length) was made along the midline of the rat’s neck to expose 2–3 cm of the right carotid artery. The proximal end of the heart was clamped with arterial forceps, and the distal end of the heart was tied with silk thread (No. 0). A V-shaped incision pointing to the heart was made at the ligation site using ophthalmic scissors. A Millar catheter (SPR-869, AD Instruments) was slowly inserted in the direction of th e heart via the right common carotid artery. Arterial waves appeared after the arterial clip was released. When the cannula entered the left ventricle, characteristic left ventricular pressure and volume waveforms would appear. Finally, a proximal spare metal wire was used to fix the catheter which was used to detect and record left ventricular pressure and volume changes, and for creating pressure–volume loops (P–V loop). Hemodynamic parameters such as SW (stroke work), CO (cardiac output), Ea (arterial elasticity), dP/dt_max_ (the maximum rate of increase in left ventricular pressure), EF (ejection fraction), Pow_max_ (maximum work), dP/dt_min_ (the maximum rate of decrease in left ventricular pressure), Tau (time constant of isovolumic relaxation) were recorded using LabChart 8 (LabChart Pro Upgrade v8 for Windows, AD Instruments). Values for Ees(ventricular end-systolic elasticity), PVA(pressure volume area), EFF (mechanical efficiency) and the slope of PRSW were measured after occlusion of blood flow at the lower vena cava.

### Real-time fluorescence quantitative PCR(q-PCR)

The manufacturer's protocol for Animal Total RNA Isolation Kit (Foregene) was used to extract and purify total RNA, using the 5 × All-In-One MasterMix (with AccuRT Genomic DNA Removal kit) cDNA synthesis kits (abm) to synthesize cDNA by reverse transcription of 2 μg of total RNA. A fully automated medical real-time PCR analysis system (Shanghai Hongshi Medical Technology Co., Ltd.) was used to perform quantitative real-time PCR in a 20 μL reaction mixture containing 10 μL 2 × qPCR MasterMix, 1.2 μL 7.5 μM gene primer mix, 2μL reverse transcription product and 6.8 μl ddH2O. The primers used to amplify the product are listed in Table 1, and GAPDH was used as a reference control for each reaction. Use a fully automated medical real-time PCR analysis system (Shanghai Hongshi Medical Technology Co., Ltd.) was used to determine the cycle-to-threshold (Ct) value of each primer pair according to the recommended guidelines. Three biologically repeated reactions were performed at each time point and primer pair.

**Table 1:** Real-time PCR primer sequences.

Gene	Forward primer	Reverse primer
GAPDH	ACAGCAACAGGGTGGTGGAC	TTTGAGGGTGCAGCGAACTT
ANP	AGCAAACTGAGGGCTCTGCT	GCTCTGGGCTCCAATCCTGT
BNP	CCAGTCTCCAGAACAATCCACGATG	GCCTTGGTCCTTTGAGAGCTGTC

### Western blot (WB)

Myocardial tissue lysate was extracted with EDTA-containing protease inhibitor (100 ×) and enhanced RIPA lysate (AR0102-30, BOSTER, 30 ml), sonicated to break down the sample, and centrifuged at 13,000 rpm for 3–5 min at 4 °C to remove debris. The supernatants were subjected to electrophoresis using homemade gel and 10% pre-cast SDS-PAGE gels (omni-EasyTM). The resolved gels from pre-cast SDS-PAGE were transferred to PVDF membranes, blocked with 5% bovine serum albumin (BSA) in Tris-buffered saline containing 0.05% Tween-20 (TBS-T) and incubated with primary antibody (MOTS-c antibody: MOTSC-101AP, FabGennix; t-AMPK antibody:AMPKα1/2, YT0216, Immunoway; p-AMPK antibody:Thr172, AF3423, Affinity.) at 4 °C overnight. The membranes were washed three times with TBS-T and incubated with HRP-conjugated secondary antibodies at room temperature for 2 h. The membrane was washed again three times, developed using UltraSignal ultra-sensitive ECL chemiluminescence substrate (Beijing 4A Biotech Co., Ltd) and imaged using the VisionWorks system (analyticjena).

### Statistical analysis

All data are expressed as mean ± standard deviation (Mean ± SD) and analyzed by SPSS25.0 software and GraphPad Prism6.0. Data were tested for normal distribution. A one-way ANOVA was used to determine the differences between groups for normally distributed data. The Kruskal–Wallis one-way ANOVA with independent samples t-tests was used for analyzing data that were not normally distributed. The significance level was set at *p* < 0.05.

## Results

### Changes of body weight and heart weight index after MOTS-c treatment during exercise training

**Figure 1:**
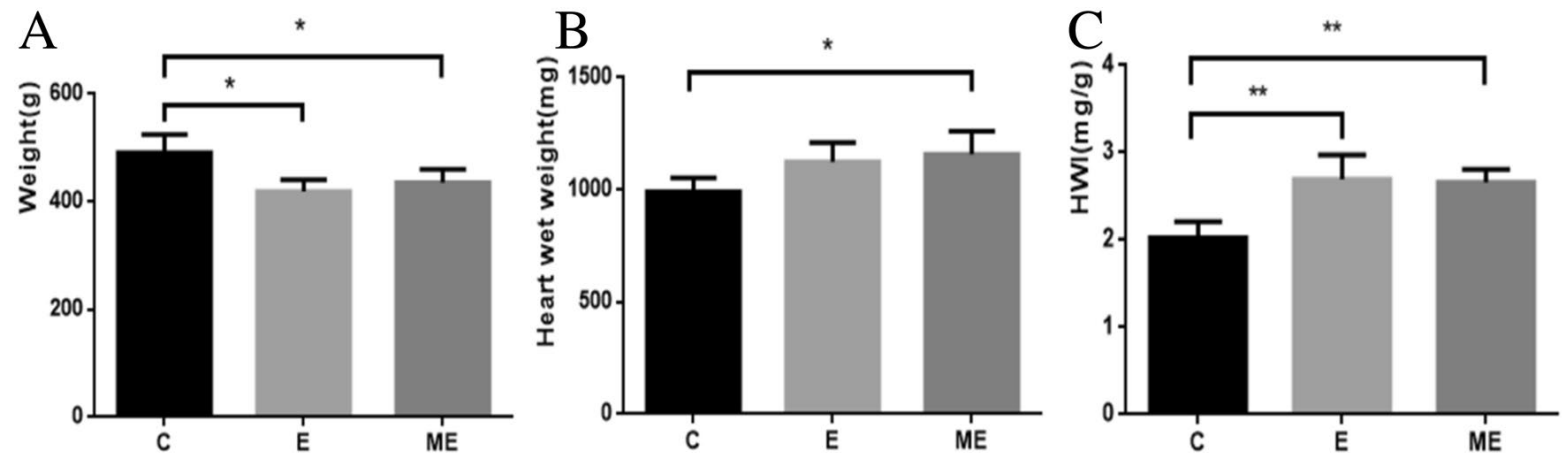
Body weight, heart wet weight and heart weight index (HWI). The changes in body weight (**A**) and heart wet weight (**B**) of rats in the control group, exercise group and MOTS-c combined exercise group were detected. Calculate the heart wet weight/body weight (HWI) ratio (**C**) to evaluate the growth response of the heart with or without MOTS-c injection during exercise. *p < 0.05, **p < 0.01, compared to group C.

The body weight (BW) of rats in groups E and ME were significantly lower than those in group C (*p* = 0.003, *p* = 0.009), and the BW of rats was similar for rats in groups ME and E (*p* = 0.359). (Fig. 1A). The wet weights for hearts from rats in groups ME (*p* = 0.015) were greater than in group C (*p* = 0.061), while wet weights were similar for hearts from rats in groups E and ME (*p* = 0.565). (Fig. 1B). The heart weight index (HWI) is a useful preliminary indicator of cardiac hypertrophy. HWI of rats in groups E (*p* = 0.001) and ME (*p* = 0.001) were greater than in group C, with no differences between HWI of rats in groups E and ME (*p* = 0.805) (Fig. 1C).

### Cardiac histology after MOTS-c treatment during exercise training

#### Hematoxylin–Eosin

**Figure 2:**
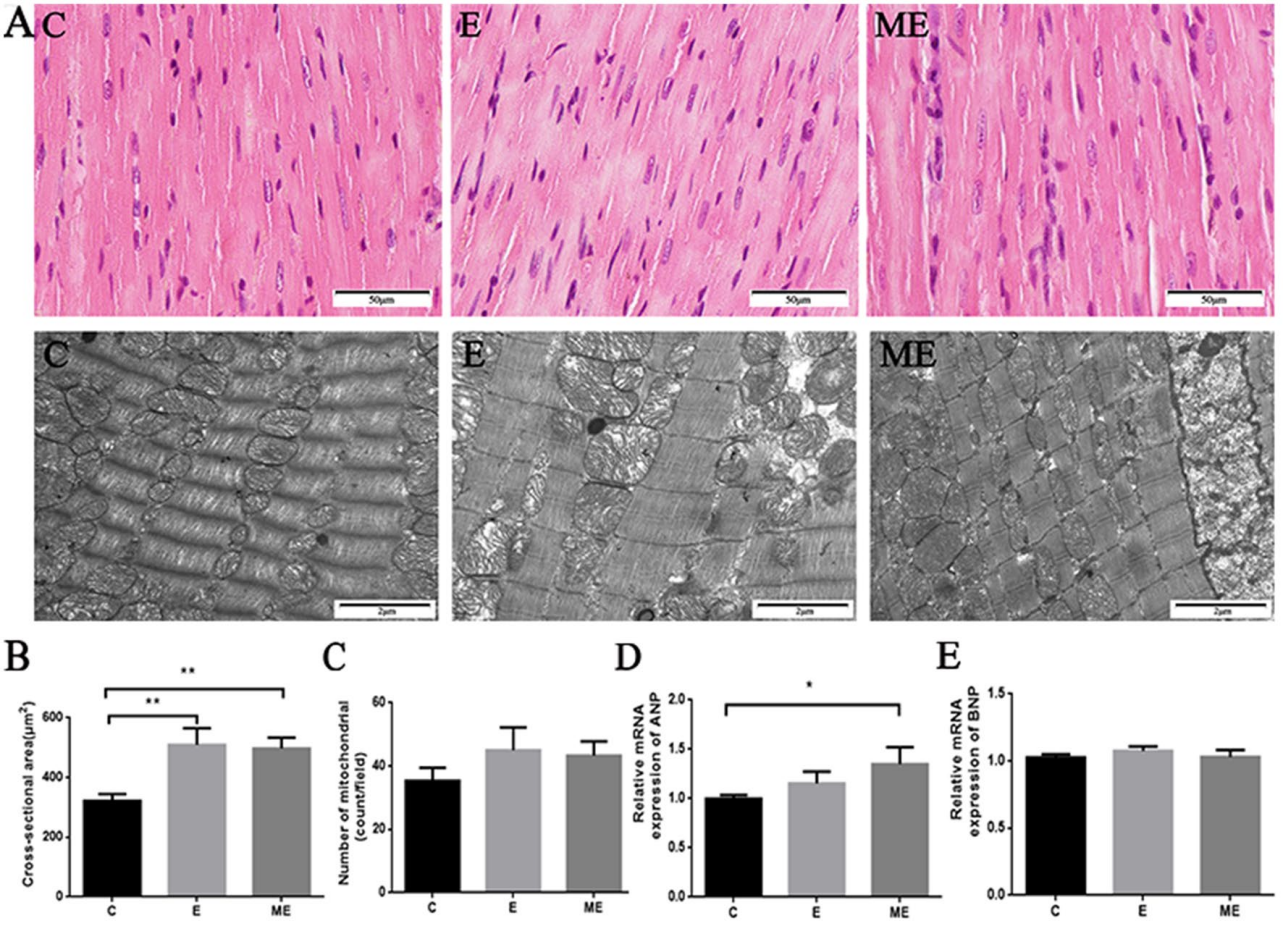
The effects of MOTS-c and exercise training on rat myocardial structure and hypertrophic genes. (**A**) is the HE staining (× 400) and TEM (× 20,000) images of the myocardium, which respectively reflect the gross and ultrastructure of the myocardium. The arrow is the lysosome. (**B**) Quantification of cross-sectional area of cardiomyocytes from H&E-stained sections. (**C**) Quantitative analysis of the number of myocardial mitochondria in TEM. (**D** and **E**) The mRNA expression of ANP and BNP. ^*^p < 0.05, ^**^p < 0.01, compared to group C. C = Control, E = Exercise training, ME = Exercise training combined with MOTS-c treatment.

Myocardial muscle fibers were continuous and complete in rats from group C, with clear structures and uniform staining, and no abnormal changes in myocardial nuclei. However, the gaps between myocardial cells were widened in rats from group E, where myocardial fibers were thickened, and myocardial cells arranged more closely. The myocardial fiber structure was clear and thickened and tended to be hypertrophied in rats from group ME, with their muscle fibers running normally with uniform staining, clear transverse striations, and no significant nuclear changes***.*** Exercise training and MOTS-c combined exercise training increased cross-sectional area (CSA) of myocardial muscle fibers compared to control group. Values for CSA in rats from groups E and ME (*p* < 0.001, *p* < 0.001) were higher than in group C, while CSA (*p* = 0.514) was similar in groups E and ME (Fig. 2A,B).

#### Transmission electron microscope

The cardiac myofibrils in rats from group C were arranged neatly, with clear light and dark bands. The mitochondria of cardiomyocytes were oval, the capsule and cristae were clear and complete. The direction of cardiac myofibrils in rats from group E were clearer, and arranged neatly and densely, but some mitochondria had broken cristae and lysosomes appeared near individual mitochondria. The myofibrils in hearts from group ME were neatly arranged, cardiac myofibrils were increased in number, with alternating light and dark bands, clear structures, complete mitochondrial membranes. There were no differences in number of myocardial mitochondria between group C and group E, ME (*p* = 0.074, *p* = 0.123). The number of cardiac mitochondria was similar in groups E and ME (*p* = 0.722). However, there was a (non-significant) trend for an increased number of mitochondria in groups E and ME (Fig. 2A,C).

In addition, values for ANP mRNA and BNP mRNA in rats from group E (*p* = 0.177, *p* = 180) were not ssignificantly changed compared to group C. ANP mRNA in group ME was greater than that in group C(*p* = 0.013), but there was no difference in BNP mRNA(*p* = 0.926). Values for ANP mRNA and BNP mRNA (*p* = 0.102, *p* = 205) were similar in groups E and ME (Fig. 2D,E).

### Changes of cardiac structure and function after MOTS-c treatment during exercise training

**Figure 3:**
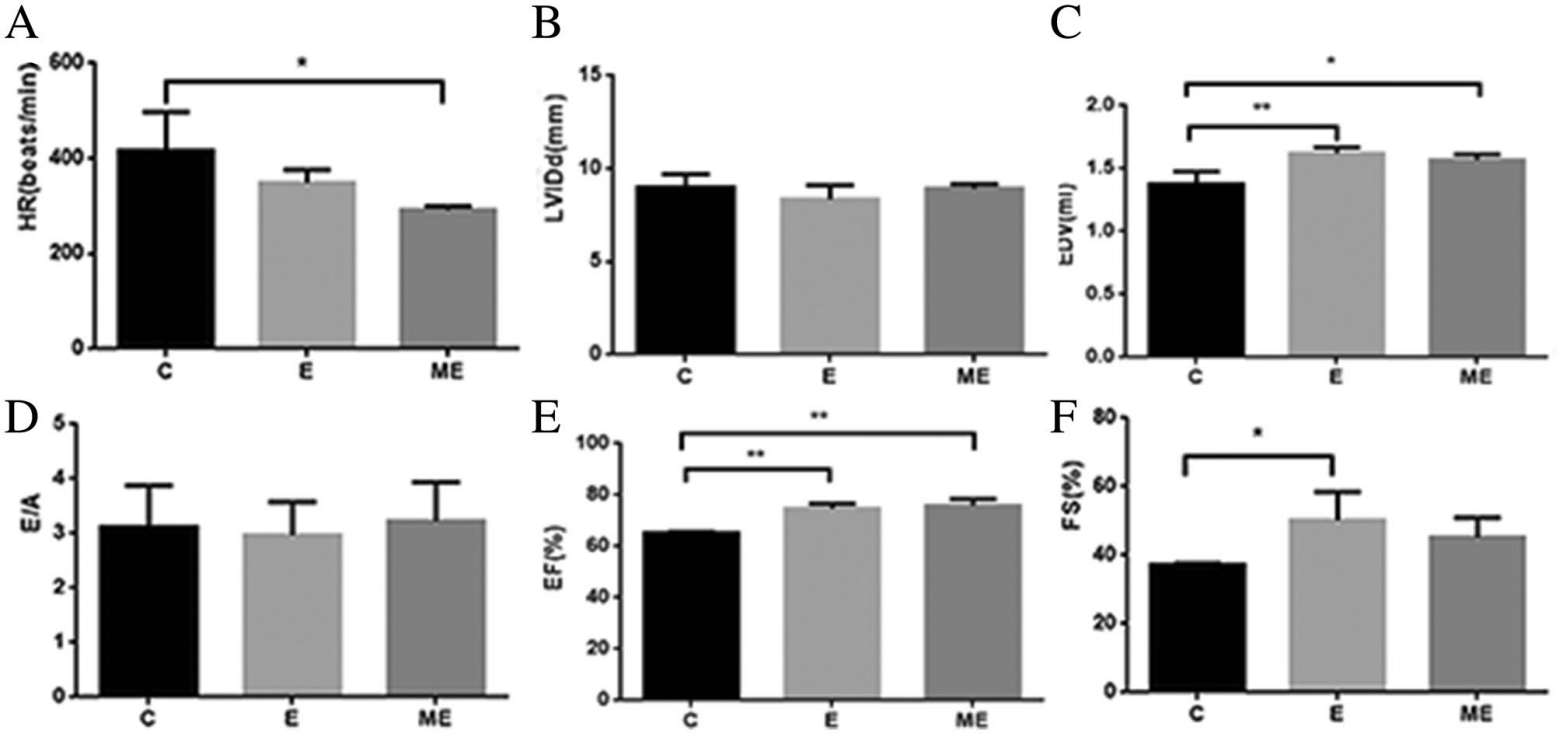
Changes of cardiac structure and function after MOTS-c. (**A**) The HR in group ME was lower than in group C. (**B**) There were no differences in LVIDd among these three groups; (**C**) The EDV in group E and ME was higher than in group C; (**D**)There were no differences in E/A among these three groups; (**E**) The EF in group E and ME was significantly higher than in group C; (**F**) The FS in group E was higher than in group C. ^*^p < 0.05, ^**^p < 0.01, compared to group C. ^#^*p* < 0.05, compared to group E.

**Figure 4:**
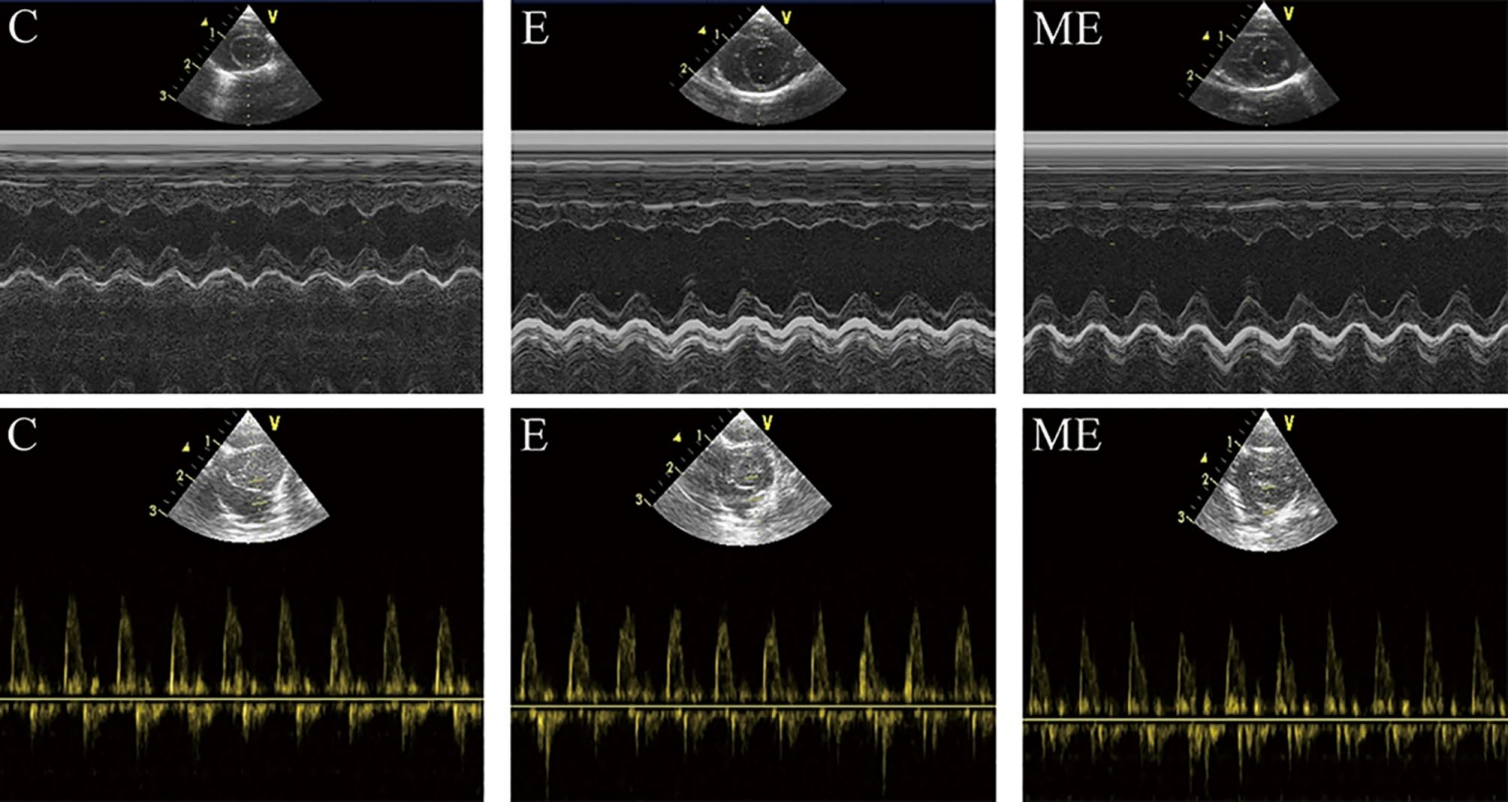
Changes of M-mode echocardiogram. The blood flow Doppler test records the ratio (E/A) of the peak blood flow velocity E in early diastole and the peak blood flow velocity A in late diastole.

Values for EDV(*p* = 0.005), EF (*p* = 0.001) and FS (*p* = 0.033) in group E were higher than that in group C, but with no differences in HR, LVIDd and E/A between groups E and C (*p* = 0.146, *p* = 0.256, *p* = 0.796). The HR in group ME was lower than in group C (*p* = 0.021), while EDV and EF in group ME was higher than in group C (*p* = 0.015, *p* < 0.001), with no differences in LVIDd (*p* = 0.691), E/A (*p* = 0.861) and FS (*p* = 0.142). There were no differences in HR (*p* = 0.204), LVIDd(*p* = 0.133), EDV(*p* = 0.370), E/A (*p* = 0.667), EF (*p* = 0.509), FS (*p* = 0.374) between groups E and ME. (Figs. 3 and 4).

### Changes in cardiac hemodynamics after MOTS-c

Carotid artery cannulation is routinely used to evaluate left ventricular systolic and diastolic functions. The left ventricular P–V loop is regarded as the most rigorous method for assessment of cardiac function^20^. The P–V loop, which is widely used in studies of human and animal cardiac performance, is the only technique that measures left ventricular function without load conditions^21–23^. Left ventricular intubation was used to evaluate the ability of MOTS-c treatment to modify changes in cardiac hemodynamics in rats due to exercise training.

#### Baseline hemodynamic data

Baseline measures of hemodynamic data includes indices of cardiac systolic function such as SW, CO, EF, dP/dtmax and Powmax. The indices of diastolic function includes dP / dtmin and Tau. Ea (arterial elasticity) integrates key elements of arterial load, including peripheral vascular resistance, common arterial compliance, characteristic impedance, systolic and diastolic time intervals^24^.

**Figure 5:**
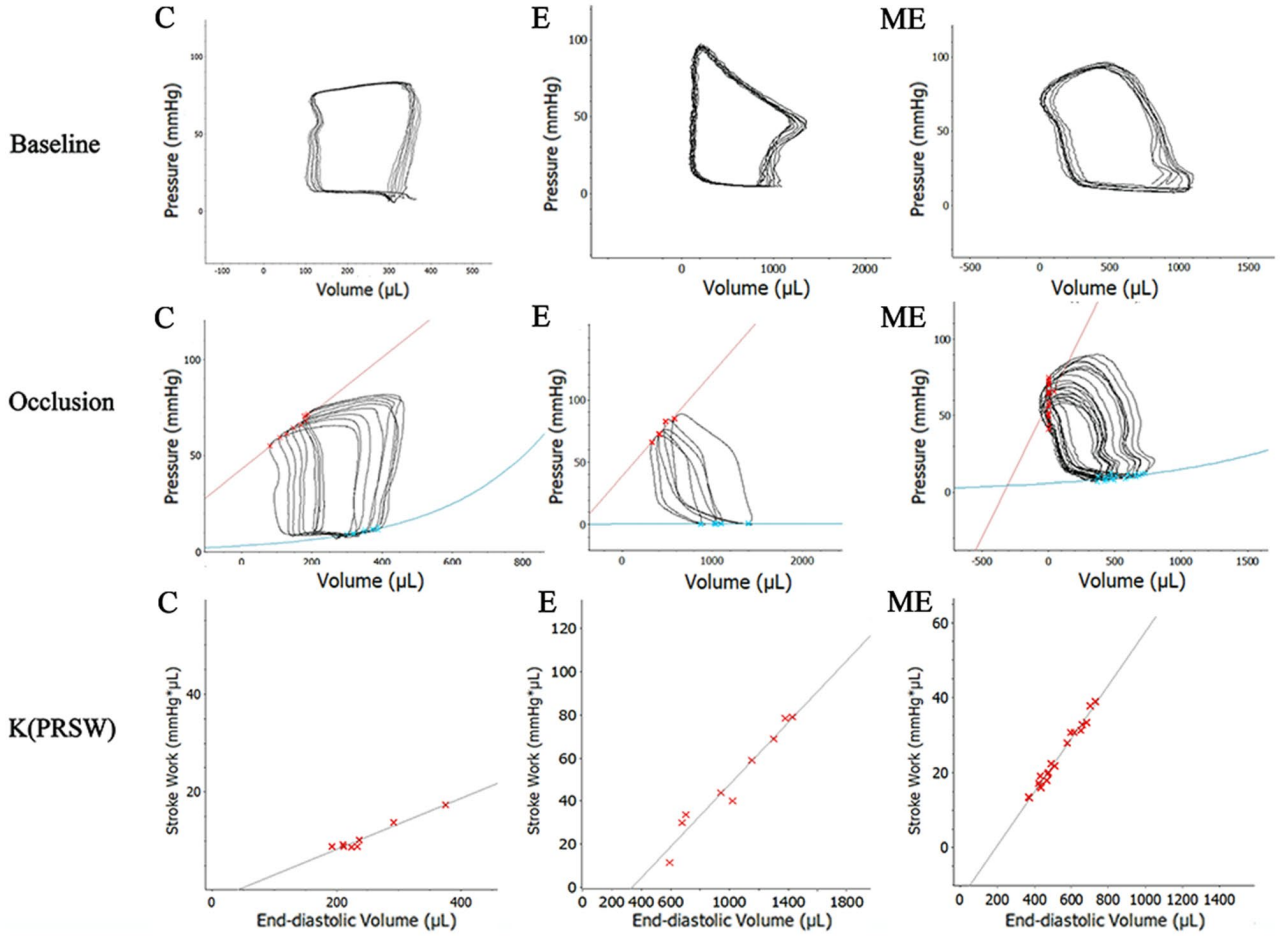
Changes in cardiac hemodynamics after MOTS-c treatment during exercise training. Baseline P–V-loops and after blood flow in the inferior vena cava blood flow was obstructed. Stroke work and the slope of ESPVR (C) [Ees (end-systolic elastance)] in the E and ME were significantly higher than in group C. (C = Control, E = Exercise training, ME = Exercise training combined with MOTS-c treatment).

Values for SW (*p* < 0.001) , CO (*p* < 0.001), EF (*p* = 0.003) and dP/dt_max_ (*p* < 0.001) in rats from groups E and ME were higher than in group C. Pow_max_ (*p* = 0.002 and dP/dt_min_ (*p* < 0.001) in group E was also higher than in group C. Ea (*p* < 0.001)in groups E and ME were lower than in group C, while there were no differences in Tau between group C and E (*p* = 0.693). EF in group ME was greater (*p* = 0.005), while values for Pow_max_ (*p* = 0.004), dP/dt_min_ (*p* = 0.004) and Tau (*p* < 0.001) were lower than in group C. Values for SW (*p* < 0.001), CO (*p* < 0.001), Pow_max_ (*p* < 0.001), dP /dt_min_ (*p* < 0.001) and Tau (*p* < 0.001) in group ME were lower than in group E, while EF (*p* = 0.639), Ea (*p* = 0.064) and dP/dt_max_ (*p* = 0.104) was similar in groups E and ME (Table 2, Fig. 5).

**Table 2:** Changes in cardiac baseline hemodynamics after MOTS-c treatment during exercise training.

Indicator	Group C	Group E	Group ME
SW(mmHg·μL)	10,596.7 ± 95.0	192,566.7 ± 5163.7**	72,316.7 ± 2355.2**^##^
CO (μL/min)	97,720.0 ± 1766.7	527,100.0 ± 12,802.0**	293,666.7 ± 2608.3**^##^
EF (%)	62.07 ± 0.88	71.58 ± 2.32**	72.64 ± 3.85**
Ea (mmHg/μL)	0.5274 ± 0.0056	0.0943 ± 0.0122**	0.0791 ± 0.0050**
Pow_max_ (mmHg·μL/s)	335,366.7 ± 35,355.7	612,166.7 ± 99,006.6**	101,670.0 ± 35,880.0**^##^
dP/dt_max_ (mmHg/s)	4263.3 ± 155.8	9038.3 ± 57.0**	9263.3 ± 185.9**
dP/dt_min_ (mmHg/s)	6686.0 ± 61.0	8323.7 ± 10.5**	6490.7 ± 67.6**^##^
Tau (ms)	12.27 ± 0.21	11.99 ± 1.39	7.27 ± 0.35**^##^

*p < 0.05, **p < 0.01, compared to group C. #*p* < 0.05, ##*p* < 0.01, compared to group E. (C = Control, E = Exercise training, ME = Exercise training combined with MOTS-c treatment. SW = stroke work, CO = cardiac output, SV = stroke volume, EF = ejection fraction, Ea = arterial elasticity, Pow_max_ = maximum work, dP/dt_max_ = the maximum rate of increase in left ventricular pressure, dP/dt_min_ = the maximum rate of decrease in left ventricular pressure, Tau = time constant of isovolumic relaxation).

#### Changes in hemodynamics after obstructing blood flow in the inferior vena cava

ESPVR (Ees) is an index of ventricular contraction that is not sensitive to cardiac load, although Ees is influenced by changes in the geometry of the left ventricle and other diastolic factors. The slope of PRSW represents the linear relationship between SW and EDV, and is independent of the size and quality of the chamber, is not affected by pre- and pro- loads but is sensitive to the systolic function of the left ventricle^25^. In addition, the mechanical efficiency (EFF) of the heart is calculated using a ratio of SW(total external mechanical work performed by the left ventricle during a single cardiac cycle) /PVA (pressure volume area)^26,27^.

Values for SW (*p* = 0.013) and the slopes for PRSW (*p* = 0.003) and PVA (*p* = 0.007) were greater in group E compared to group C, but there were no differences in Ees (*p* = 0.113), Ea/Ees (*p* = 0.061) and EFF (*p* = 0.908) between groups E and C. EFF in group ME was higher than in group C (*p* = 0.032) with an increased slope of PRSW (*p* < 0.001). There were no significant differences in Ees (*p* = 0.113), Ea/Ees (*p* = 0.072), SW (*p* = 0.068), and PVA (*p* = 0.748) between groups ME and C. Moreover, values for PVA (*p* = 0.010), EFF (*p* = 0.038) and the slope of PRSW (*p* = 0.001) were higher in group ME than group E, with no significant differences in Ees (*p* = 0.113), Ea/Ees (*p* = 0.915) and SW (*p* = 0.251) between ME and E (Table 3, Fig. 5).

**Table 3:** Changes in hemodynamics after obstructing blood flow in the inferior vena cava.

Indicator	Group C	Group E	Group ME
Ees (K)	0.00433 ± 0.00306	0.00464 ± 0.00335	0.009520 ± 0.01395
PVA (mmHg·µL)	26,250.0 ± 15,228.55	88,800.0 ± 70,546.68**	32,813.33 ± 10,023.80*#*
EFF (%)	29.86 ± 8.95	31.43 ± 9.68	66.17 ± 24.41*#
Slope of PRSW	42.66 ± 8.03	70.98 ± 4.91**	102.69 ± 8.62**##

*p < 0.05, **p < 0.01, compared to group C. #*p* < 0.05, ##*p* < 0.01, compared to group E. (C = Control, E = Exercise training, ME = Exercise training combined with MOTS-c treatment. Ees = ventricular end-systolic elasticity, PVA = pressure volume area, EFF = mechanical efficiency, the slope of PRSW = K(SW − EDV)).

### Exogenous injection of MOTS-c during exercise can increase the endogenous MOTS-c of the myocardium and activate AMPK

**Figure 6:**
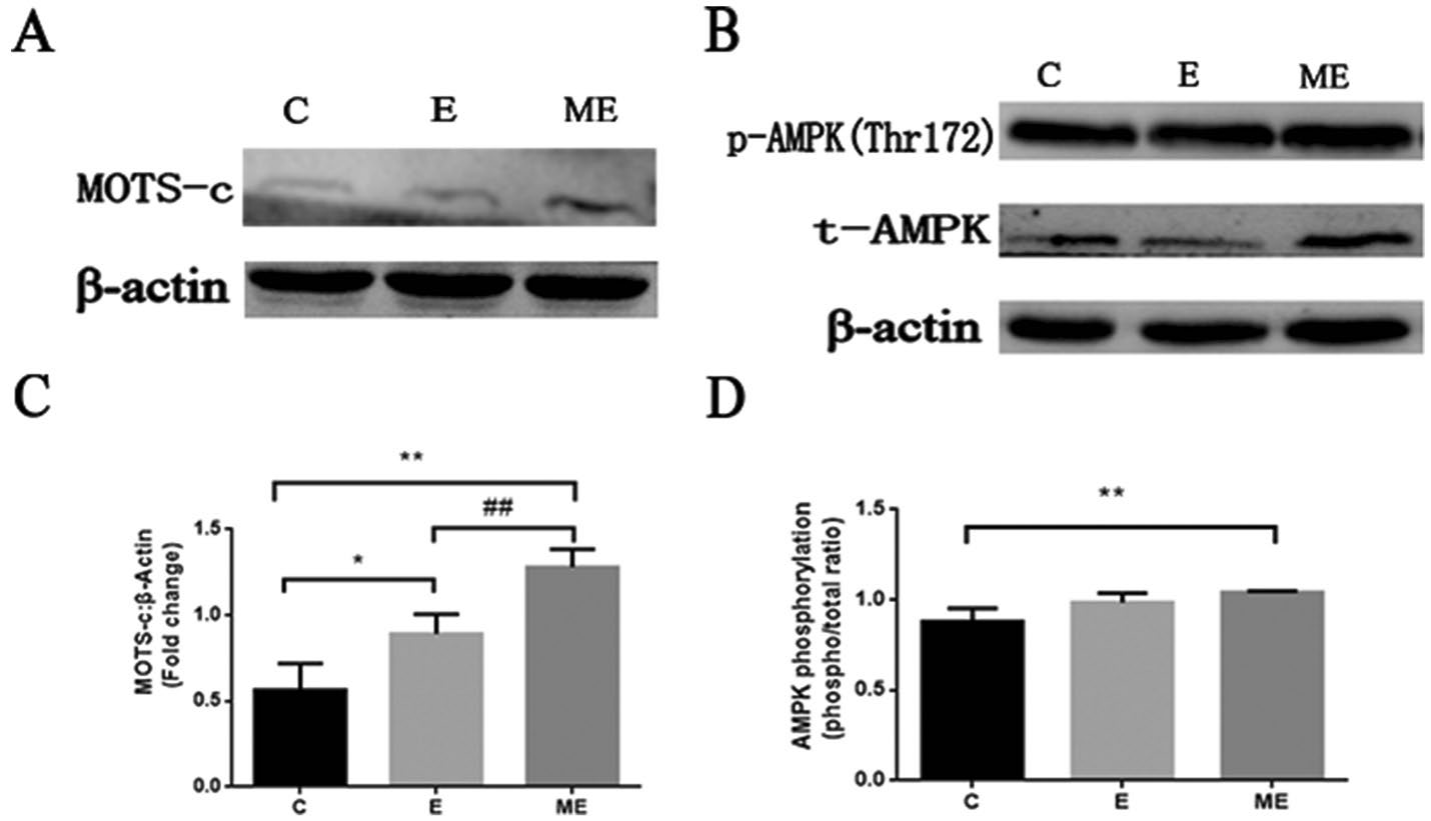
Changes in the protein expression of MOTS-c, p-AMPK, and t-AMPK in each group of rats. (**A** and **B**) are the representative images of MOTS-c, p-AMPK(Thr172), t-AMPK. (**C**) is the change of MOTS-c compared to β-Actin multiple. (**D**) is stoichiometric AMPK phosphorylation (phosphor/total ratio). *p < 0.05, **p < 0.01, compared to group C. ^#^*p* < 0.05, ^##^*p* < 0.01, compared to group E. (C = Control, E = Exercise training, ME = Exercise training combined with MOTS-c treatment).

The content of MOTS-c in groups E and ME were higher (*p* = 0.020, *p* < 0.001) than in group C, and the levels of MOTS-c of group ME after exogenous injection of MOTS-c was also significantly higher than that of group E (*p* = 0.009). Levels of p-AMPK in group ME was significantly increased compared to group C (*p* = 0.021), while there were no changes in group E (*p* = 0.108). There was no difference in p-AMPK between groups E and ME (*p* = 0.270). There were no significant differences in values for t-AMPK between the group C and group E, ME (*p* = 0.833, *p* = 0.583), and with no differences between the groups E and ME (*p* = 0.731). AMPK phosphorylation (phosphor/total) in group ME was significantly higher than that in group C (*p* = 0.009), while there were no significant differences in AMPK phosphorylation among group C, E and ME (E vs. C, *p* = 0.051; E vs. ME, *p* = 0.233) It seems that exercise combined with MOTS-c treatment activate AMPK, instead of increasing total AMPK protein expression (Fig. 6).

## Discussion

Long-term moderate exercise training can improve cardiac function, reduce the incidence of cardiovascular diseases, and delay aging. Aerobic exercise promotes physiological remodeling of the myocardium, causing cardiac enlargement by 10–20%^28^. Endurance athletes and those who engage in regular fitness develop beneficial cardiac structural and functional changes. For example, left ventricular end-diastolic diameter and left ventricular wall thickness are increased, as are the ejection fraction and short axis shortening rate^29^.

We examined the effects of MOTS-c on cardiac histopathology, echocardiography, and P–V-loop tests in rats after 12-weeks of exercise training. Aerobic exercise combined with MOTS-c and aerobic exercise alone caused cardiac hypertrophy induced by physiological remodeling. Importantly, myocardial mechanoenergetics and enhanced cardiac function was greater in exercised rats receiving MOTS-c compared to untreated rats.

### Effects of aerobic exercise combined with MOTS-c treatment on body weight and heart weight index (HWI)

Aerobic exercise can induce physiological myocardial hypertrophy, increase the expression of some myocardial energy metabolism proteins, stimulate myocardial energy metabolism, and enhance myocardial contractility and pumping function^30^. For example, 8 weeks of aerobic exercise decreased body weight and increased HWI in rats^31^, while rats exposed to swimming training for 12 weeks had decreased body weights and increased heart weight and HWI^32^. Supplementation with MOTS-c induces hypoglycemia and a reduction in lipid levels^33–35^. We reported that exercised rats reduced body weights and increased HWI, consistent with other reports that aerobic exercise induced myocardial hypertrophy. Exercised rats treated MOTS-c had similar body weights and HWI compared to untreated exercised rats, suggesting that there may be an synergetic effects between MOTS-c and aerobic exercise to promote physiological myocardial hypertrophy.

### Effects of aerobic exercise combined with MOTS-c treatment on myocardial morphology

We assessed the effects of aerobic exercise combined with MOTS-c treatment on myocardial structure using HE staining and transmission electron microscopy. Aerobic exercise (12 weeks) reversed collagen deposition, reduced the number of disordered cells, and restored myocardial cell structure in rats fed a high-fat diet^35,36^. We demonstrate that exercise widened intercellular myocardial cell spaces and caused myocardial fibers to be thickened and more closely arranged. Both aerobic exercise and aerobic exercise combined with MOTS-c treatment induced myocardial hypertrophy, but myocardial hypertrophy caused by aerobic exercise combined with MOTS-c group was more distinct, suggesting that MOTS-C might play an important role in regulating physiological hypertrophy of the myocardium. Our quantitative statistics to determine the cross-sectional area of muscle fibers in the longitudinal section of the myocardium, indicated that CSA increased after exercise training and exercise combined with MOTS-c intervention. This finding is similar to that reported by Medina et al. where the cross-sectional area of cardiomyocytes increased in C57/B16 mice undergoing voluntary running^37^.

Other studies also indicated that aerobic exercise alters cardiac ultrastructure. Campos et al. reported that aerobic exercise training after myocardial infarction in rats re-established the balance of myocardial mitochondrial fission and fusion, and also improved myofibrillar arrangement^38^. Our results show that cardiac ultrastructure of exercised rats was slightly damaged. Important is our finding that treatment with MOTS-c during exercise training restored mitochondrial structure and the removal of lysosomes, suggesting that MOTS-c treatment could protect the myocardium and avoid ultrastructural damage. Mitochondria supply energy during myocardial contractility, there were no increases in mitochondrial numbers the groups E and ME, but there was a trend for increases, indicating that the energy supply was perhaps improving. Other studies also used the number of mitochondria to reflect the dynamics and function of the myocardium^39,40^.

ANP and BNP are markers of cardiac hypertrophy. Changes in plasma ANP mRNA levels cannot fully reflect the effects of exercise on the synthesis and secretion of ANP in cardiomyocytes^41^. Therefore, we tested the myocardial ANP and BNP mRNA and found that the ANP mRNA in the ME group was significantly increased, and that ANP mRNA levels in rats from group E tended to increase, but with was no changes in BNP mRNA levels. The Libonati team conducted a 12-week treadmill exercise intervention on Wistar rats and reported no changes in ANP mRNA in the exercise and the sedentary groups^42^, which is consistent with the results of our study. In addition, BNP levels did not change significantly in exercised rats^43^, similar to the results of our study as well. These findings indicate that MOTS-c can promote myocardial hypertrophy.

### Effect of aerobic exercise combined with MOTS-c on cardiac function

Endurance exercise can induce a unique cardioprotective phenotype, with cardiac hypertrophy promoted by aerobic exercise being a well-studied adaptation^44^. This adaptation normalizes ventricular wall stress, reduces oxygen consumption, increases work capacity, and has some mechanical advantages^45^.

#### Systolic function

Exercise induces changes in left ventricular systolic function^46^. Our study indicates that indicators of systolic function (such as SW, CO, Pow_max_, dP/dt_max_, EF, and FS) increased after 8-week exercise training in rats. Measures of EF reflected changes in hemodynamics, and is supported by studies in rats undergoing swimming training for 12 weeks, where changes of systolic function indexes^47^ were similar to our findings that 12 weeks of aerobic exercise increased cardiac systolic function.

Ees of rats participating in swimming training was significantly increased, indicating improvements in the contractile state of the LV myocardium^48^. The findings of the current study indicate no changes in Ees after 12 weeks of treadmill exercise, while Ees trended to increase in exercising rats after treatment with MOTS-c, suggesting that MOTS-c improved the systolic function of rats. Our results show that the slope of PRSW was increased in exercised rats, which is consistent with the results of Radovits et al.^25^. Treatment with MOTS-c increased the slope of PRSW in exercised rats, indicating that MOTS-c modulates myocardial contraction.

#### Diastolic function

We show that 12 weeks exercise training impacted diastolic function in rats, although LVIDd, E/Aand Tau did not change, while dP/dtmin, EDV were all increased by exercise. Of interest is that MOTS-c, and combining MOTS-c with exercise reduced Tau, indicating that MOTS-c can improve the diastolic function of the heart to some extent. The decrease in the dP/dtmin of group ME compared to the group E may be due to the different effects and sensitivity of MOTS-c on various diastolic function parameters.

#### Mechanical efficiency

The P–V-loop was used to analyze mechanical energy changes related to myocardial remodeling. Ees and Ea measure left ventricular contractility and vascular load index relatively independently of load^49^. We report that Ea decreased after 12-weeks of aerobic exercise training, as also reported by others^24^. The increased Ees (myocardial contractility) and the decreased Ea reduced the ventricular-arterial coupling ratio of exercised rats. The Ea/Ees ratio determines overall cardiac performance. The enhanced ventricular-arterial coupling we report reflects improved matching between the left ventricle and the systemic arterial circulationin in exercised rats, so allowing for efficient transfer of blood from the left ventricle to the periphery without excessive pressure changes^50^. In our experiments, the Ea/Ees ratio tended to be reduced after exercise alone and also after exercise combined with MOTS-c treatment, indicating that exercise and MOTS-c could modulate the coupling of the left ventricle and the arterial system.

We report that exercise increased EFF, indicating that exercise-induced cardiac hypertrophy was able to optimize metabolic efficiency, increase mechanical workload, and reduce cardiomyocyte oxygen consumption, confirming a previous report that aerobic exercise improved cardiac mechanical efficiency^50^. Our data indicates that treatment with MOTS-c improved the mechanical efficiency of aerobic exercise-induced myocardial hypertrophy, suggesting that MOTS-c may have a unique role in improving myocardial mechanical efficiency.

### Exogenous injection of MOTS-c during exercise can increase the endogenous MOTS-c of the myocardium and activate AMPK

The findings of Lee et al. indicated that mice had higher levels of MOTS-c than humans^12^. They reported that levels of MOTS-c were increased in the myocardium, skeletal muscle and brain, and that exercised endogenous levels of MOTS-c. Another study by Reynolds et al. reported that levels of endogenous MOTS-c in humans increased by 1.5 times after exercise, which is consistent with the results of our study. Surprisingly, after we injected MOTS-c into exercise-trained rats, the endogenous MOTS-c rose more robustly, indicating that exogenous was transformed into endogenous MOTS-c, confirming the finding of Kim et al. that endogenous MOTS-c increased after MOTS-c treatment^52^. We also found that increased levels of endogenous MOTS-c in the myocardium in rats from the ME group activated AMPK and increased phosphorylated AMPK, while the total amount remained unchanged. Activated AMPK regulates other proteins to improve cardiac performance. A study by Lee et al. also found that p-AMPK increased after MOTS-c treatment, while t-AMPK remained unchanged^12^. Our study indicates that administering exogenous MOTS-c increases endogenous levels of MOTS-c of the myocardium, which in turn slightly activates AMPK.

## Study limitations

Firstly, our study is limited to young male rats, and should be expanded to include differences related to gender, age, and other species. Secondly, we did not investigate the mechanistic profile of the effects of MOTS-c on myocardial hypertrophy. Finally, MOTS-c injection slightly activated AMPK, but AMPK protein expression remained unchanged. It suggested that AMPK, which as a possible mechanism, could play a modulatory role. Thus, further research is necessary to understand the prominent mechanism of cardiac function improvement induced by MOTS-c.

## Conclusion

Administration of exogenous MOTS-c increases endogenous levels of myocardial MOTS-c, improves the mechanical efficiency of the myocardium, strengthens the systolic function of the heart, and helps to improve diastolic function during exercise training. Our finding provide an experimental basis for the use of putative exercise supplements to further promote the cardiovascular benefits of exercise training.

## Supplementary Information

Supplementary Information 1.

Supplementary Information 2.

Supplementary Information 3.

Supplementary Information 4.

Supplementary Information 5.

Supplementary Information 6.

## References

[1] Verdoorn KS, Matsuura C, Borges JP (2017). Exercise for cardiac health and regeneration: Killing two birds with one stone. Ann. Transl. Med..

[2] Sanchis-Gomar F, Fiuza-Luces C, Lucia A (2015). Exercise as the master polypill of the 21st century for the prevention of cardiovascular disease. Int. J. Cardiol..

[3] Tao L, Bei Y, Zhang H, Xiao J, Li X (2015). Exercise for the heart: Signaling pathways. Oncotarget.

[4] Bernardo BC, Ooi JYY, Weeks KL, Patterson NL, McMullen JR (2018). Understanding key mechanisms of exercise-induced cardiac protection to mitigate disease: Current knowledge and emerging concepts. Physiol. Rev..

[5] Schüttler, D., Clauss, S., Weckbach, L. T. & Brunner, S. Molecular mechanisms of cardiac remodeling and regeneration in physical exercise. *Cells* **8**, (2019). 10.3390/cells8101128 PMC6829258 31547508

[6] Bo, B. *et al.* The molecular mechanisms associated with aerobic exercise-induced cardiac regeneration. *Biomolecules* **11**, (2020). 10.3390/biom11010019 PMC7823705 33375497

[7] Marini M (2007). Mild exercise training, cardioprotection and stress genes profile. Eur. J. Appl. Physiol..

[8] Li J-Y, Pan S-S, Wang J-Y, Lu J (2019). Changes in autophagy levels in rat myocardium during exercise preconditioning-initiated cardioprotective effects. Int. Heart J..

[9] Li H (2016). Acute exercise-induced mitochondrial stress triggers an inflammatory response in the myocardium via NLRP3 inflammasome activation with mitophagy. Oxid. Med. Cell Longev..

[10] Ghahremani R, Damirchi A, Salehi I, Komaki A, Esposito F (2018). Mitochondrial dynamics as an underlying mechanism involved in aerobic exercise training-induced cardioprotection against ischemia-reperfusion injury. Life Sci..

[11] Nashine, S. & Kenney, M. C. Effects of mitochondrial-derived peptides (MDPs) on mitochondrial and cellular health in AMD. *Cells* **9**, (2020). 10.3390/cells9051102 PMC7290668 32365540

[12] Lee C (2015). The mitochondrial-derived peptide MOTS-c promotes metabolic homeostasis and reduces obesity and insulin resistance. Cell Metab..

[13] Li S, Laher I (2015). Exercise pills: At the starting line. Trends Pharmacol. Sci..

[14] Li S, Laher I (2017). Exercise mimetics: Running without a road map. Clin. Pharmacol. Ther..

[15] The mitochondrial-derived peptide MOTS-c is a regulator of plasma metabolites and enhances insulin sensitivity—PubMed. https://pubmed.ncbi.nlm.nih.gov/31293078/. 10.14814/phy2.14171 PMC6640593 31293078

[16] Yang Y (2019). The role of mitochondria-derived peptides in cardiovascular disease: Recent updates. Biomed. Pharmacother..

[17] Qin Q (2018). Downregulation of circulating MOTS-c levels in patients with coronary endothelial dysfunction. Int. J. Cardiol..

[18] Li H, Ren K, Jiang T, Zhao G-J (2018). MOTS-c attenuates endothelial dysfunction via suppressing the MAPK/NF-κB pathway. Int. J. Cardiol..

[19] Wei M (2020). Mitochondrial-derived peptide MOTS-c attenuates vascular calcification and secondary myocardial remodeling via adenosine monophosphate-activated protein kinase signaling pathway. Cardiorenal. Med..

[20] Pacher P, Nagayama T, Mukhopadhyay P, Bátkai S, Kass DA (2008). Measurement of cardiac function using pressure-volume conductance catheter technique in mice and rats. Nat. Protoc..

[21] Tello K (2020). Right ventricular function correlates of right atrial strain in pulmonary hypertension: A combined cardiac magnetic resonance and conductance catheter study. Am. J. Physiol. Heart Circ Physiol..

[22] Ortiz-León, G., Barrantes-Vargas, H. J., Arguedas-Sandí, M., Pacheco-Chaverri, J. Á. & Vílchez-Monge, M. An approximation of heart failure using cardiovascular simulation toolbox. *Biomimetics (Basel)* **4**, (2019). 10.3390/biomimetics4030047 PMC6784415 31295796

[23] Huang H (2019). Rolipram, a PDE4 inhibitor, enhances the inotropic effect of rat heart by activating SERCA2a. Front Pharmacol..

[24] Sunagawa K, Maughan WL, Burkhoff D, Sagawa K (1983). Left ventricular interaction with arterial load studied in isolated canine ventricle. Am. J. Physiol..

[25] Radovits T (2013). Rat model of exercise-induced cardiac hypertrophy: Hemodynamic characterization using left ventricular pressure-volume analysis. Am. J. Physiol. Heart Circ. Physiol..

[26] Yagi Y (1989). Response of cardiac patients to dynamic exercise: Analysis with ‘systolic’ pressure-volume area. J. Cardiol..

[27] Tachibana H (2005). Levosimendan improves LV systolic and diastolic performance at rest and during exercise after heart failure. Am. J. Physiol. Heart Circ. Physiol..

[28] Xiang K, Qin Z, Zhang H, Liu X (2020). Energy metabolism in exercise-induced physiologic cardiac hypertrophy. Front Pharmacol..

[29] Exercise-Induced changes in glucose metabolism promote physiological cardiac growth - PubMed. https://pubmed.ncbi.nlm.nih.gov/28860122/. 10.1161/CIRCULATIONAHA.117.028274 PMC5704654 28860122

[30] Gibb AA, Hill BG (2018). Metabolic coordination of physiological and pathological cardiac remodeling. Circ. Res..

[31] Reyes DRA (2019). Exercise during transition from compensated left ventricular hypertrophy to heart failure in aortic stenosis rats. J. Cell Mol. Med..

[32] Gazdag P (2020). Increased Ca2+ content of the sarcoplasmic reticulum provides arrhythmogenic trigger source in swimming-induced rat athlete’s heart model. Sci. Rep..

[33] Westerterp KR (2019). Exercise for weight loss. Am. J. Clin. Nutr..

[34] Lu H (2019). MOTS-c peptide regulates adipose homeostasis to prevent ovariectomy-induced metabolic dysfunction. J. Mol. Med. (Berl).

[35] Guo Q (2020). Adiponectin treatment improves insulin resistance in mice by regulating the expression of the mitochondrial-derived peptide MOTS-c and its response to exercise via APPL1-SIRT1-PGC-1α. Diabetologia.

[36] Chen X (2019). Aerobic exercise ameliorates myocardial inflammation, fibrosis and apoptosis in high-fat-diet rats by inhibiting P2X7 purinergic receptors. Front Physiol..

[37] Medina AJ (2020). Cardiac up-regulation of NBCe1 emerges as a beneficial consequence of voluntary wheel running in mice. Arch. Biochem. Biophys..

[38] Campos JC (2017). Exercise reestablishes autophagic flux and mitochondrial quality control in heart failure. Autophagy.

[39] Wen JJ, Cummins CB, Radhakrishnan RS (2020). Burn-induced cardiac mitochondrial dysfunction via interruption of the PDE5A-cGMP-PKG pathway. Int. J. Mol. Sci..

[40] Wu B (2018). TLR4 activation promotes the progression of experimental autoimmune myocarditis to dilated cardiomyopathy by inducing mitochondrial dynamic imbalance. Oxid. Med. Cell Longev..

[41] Pan SS (2008). Alterations of atrial natriuretic peptide in cardiomyocytes and plasma of rats after different intensity exercise. Scand. J. Med. Sci. Sports.

[42] Libonati JR, MacDonnell SM (2011). Cardiac β-adrenergic responsiveness with exercise. Eur. J. Appl. Physiol..

[43] Gutkowska J, Paquette A, Wang D, Lavoie J-M, Jankowski M (2007). Effect of exercise training on cardiac oxytocin and natriuretic peptide systems in ovariectomized rats. Am. J. Physiol. Regul Integr. Comput. Physiol..

[44] Lee B-A, Oh D-J (2016). The effects of long-term aerobic exercise on cardiac structure, stroke volume of the left ventricle, and cardiac output. J. Exerc. Rehabil..

[45] Ferreira R (2015). Unraveling the exercise-related proteome signature in heart. Basic Res. Cardiol..

[46] Mi C, Qin X, Hou Z, Gao F (2019). Moderate-intensity exercise allows enhanced protection against oxidative stress-induced cardiac dysfunction in spontaneously hypertensive rats. Braz. J. Med. Biol. Res..

[47] Kovács A (2015). Strain and strain rate by speckle-tracking echocardiography correlate with pressure-volume loop-derived contractility indices in a rat model of athlete’s heart. Am. J. Physiol. Heart Circ. Physiol..

[48] He Q, Cheng J, Wang Y (2019). Chronic CaMKII inhibition reverses cardiac function and cardiac reserve in HF mice. Life Sci..

[49] Hieda M (2018). Impact of lifelong exercise training dose on ventricular-arterial coupling. Circulation.

[50] Chantler PD, Lakatta EG, Najjar SS (2008). Arterial-ventricular coupling: mechanistic insights into cardiovascular performance at rest and during exercise. J. Appl. Physiol..

[51] Reynolds JC (2021). MOTS-c is an exercise-induced mitochondrial-encoded regulator of age-dependent physical decline and muscle homeostasis. Nat. Commun..

[52] Kim KH, Son JM, Benayoun BA, Lee C (2018). The mitochondrial-encoded peptide MOTS-c translocates to the nucleus to regulate nuclear gene expression in response to metabolic stress. Cell Metab..

