# Supplementary material for: The mitochondrial signaling peptide MOTS-c improves myocardial performance during exercise training in rats
Source: Sci Rep. 2021 Oct 11;11:20077. doi: 10.1038/s41598-021-99568-3 (PMC8505603; doi:10.1038/s41598-021-99568-3)

MOTS-c

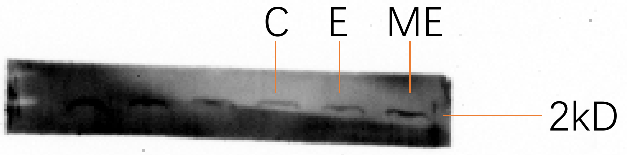

MOTS-c

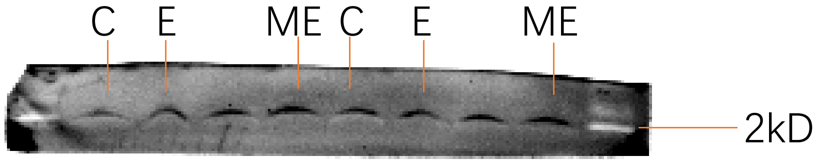

β-actin1

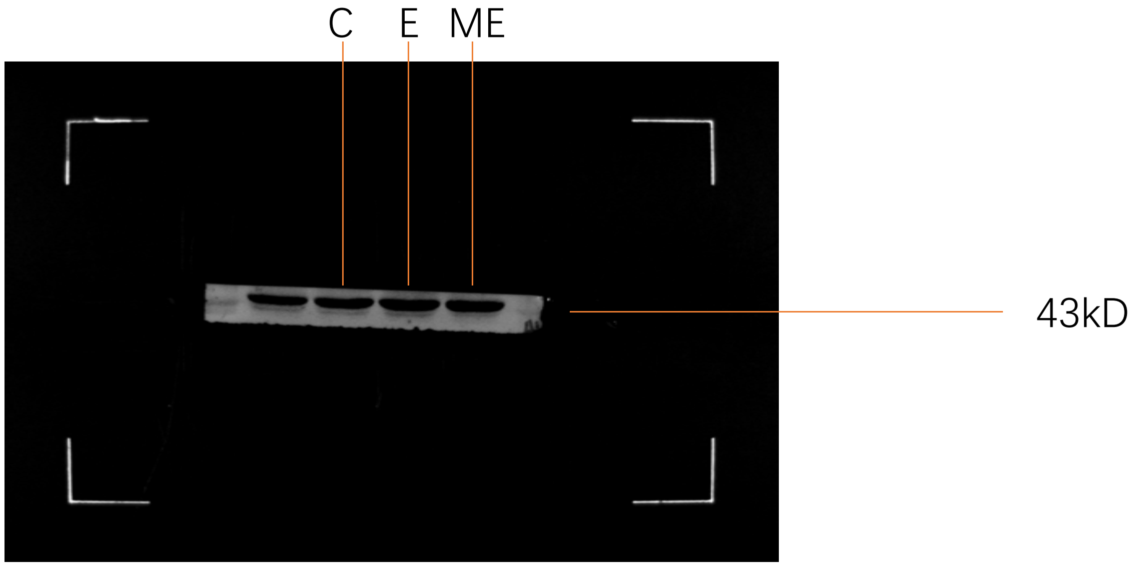

β-actin1

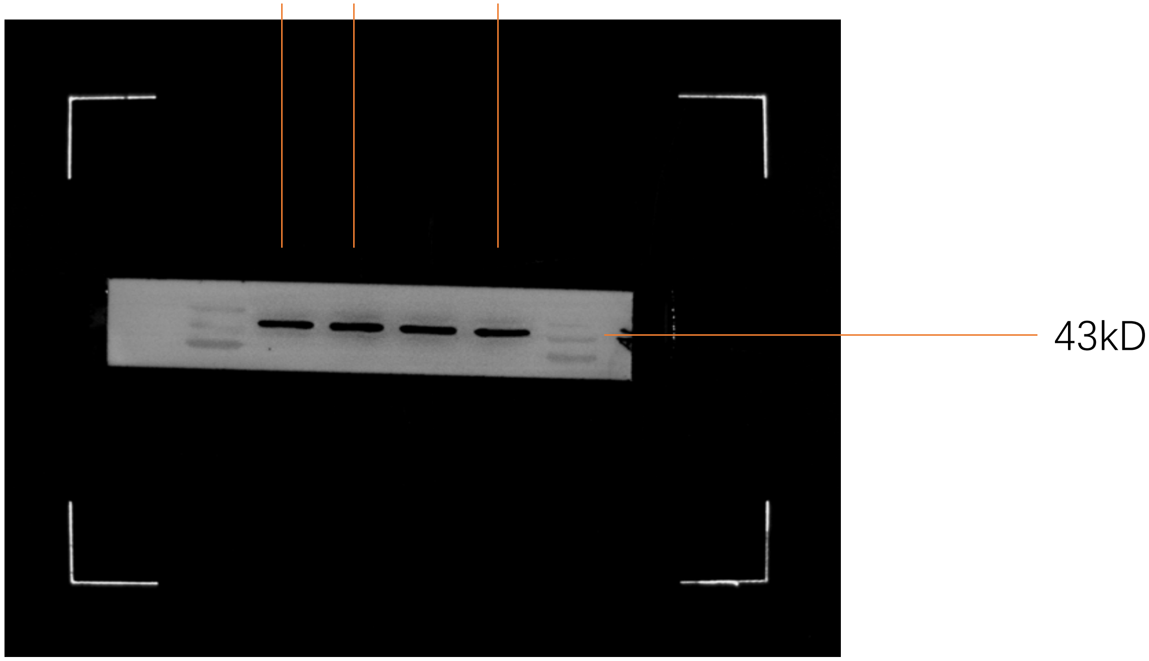

β-actin1

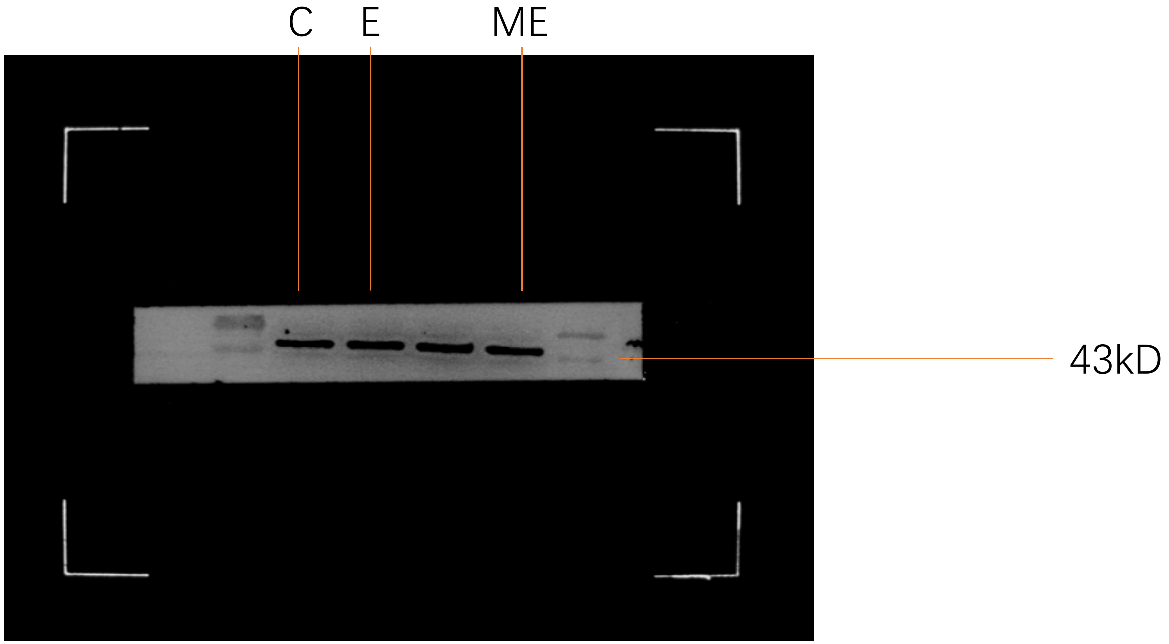

p-AMPK

C E ME

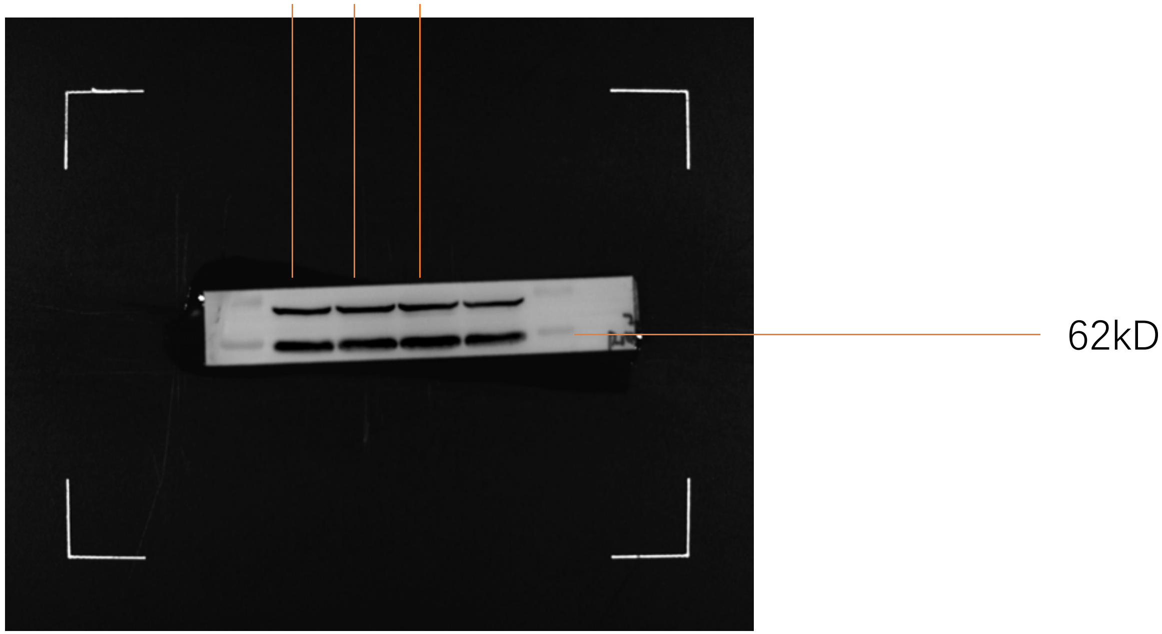

62kD

p-AMPK

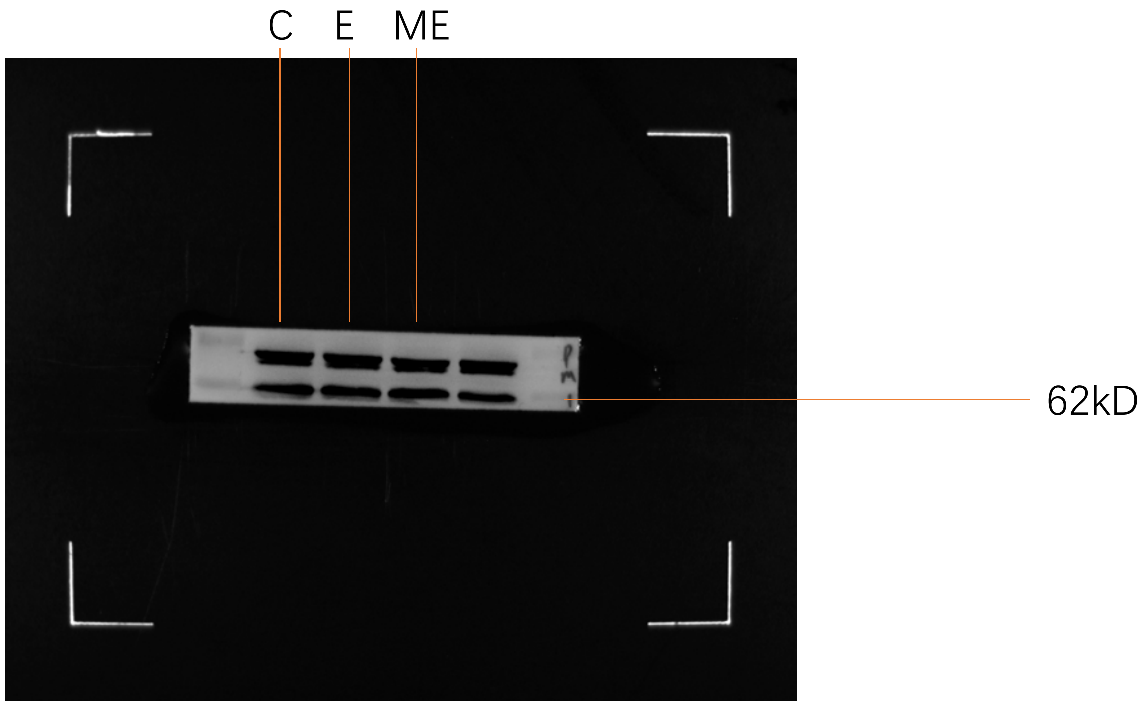

p-AMPK

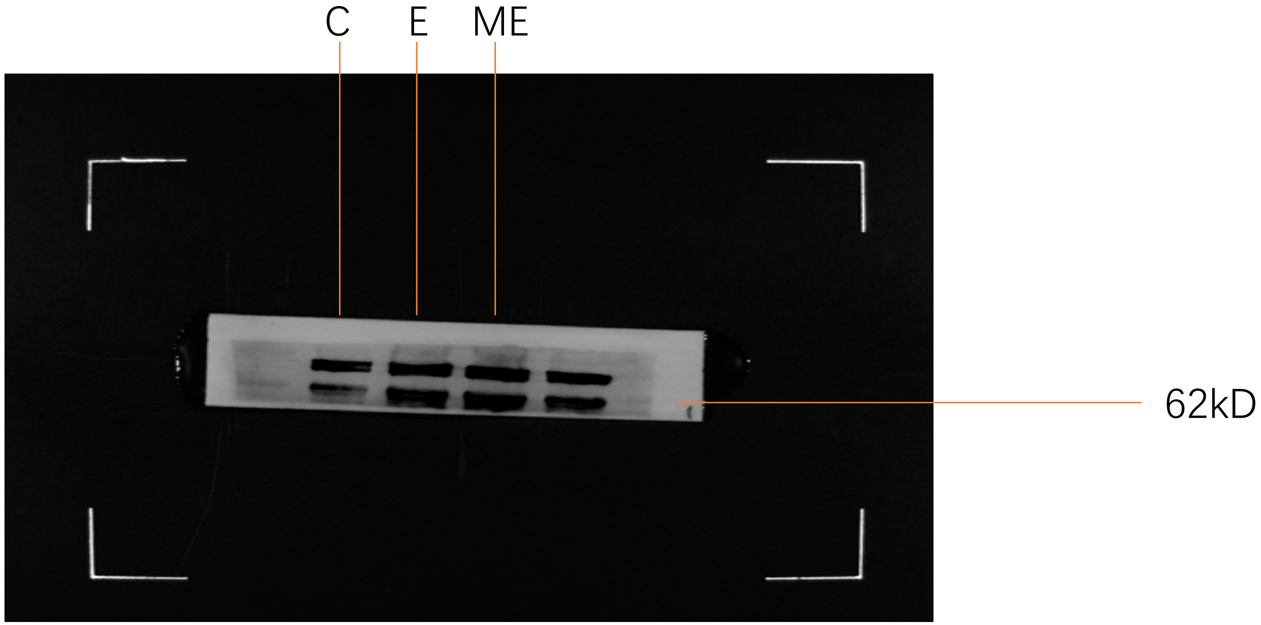

t-AMPK

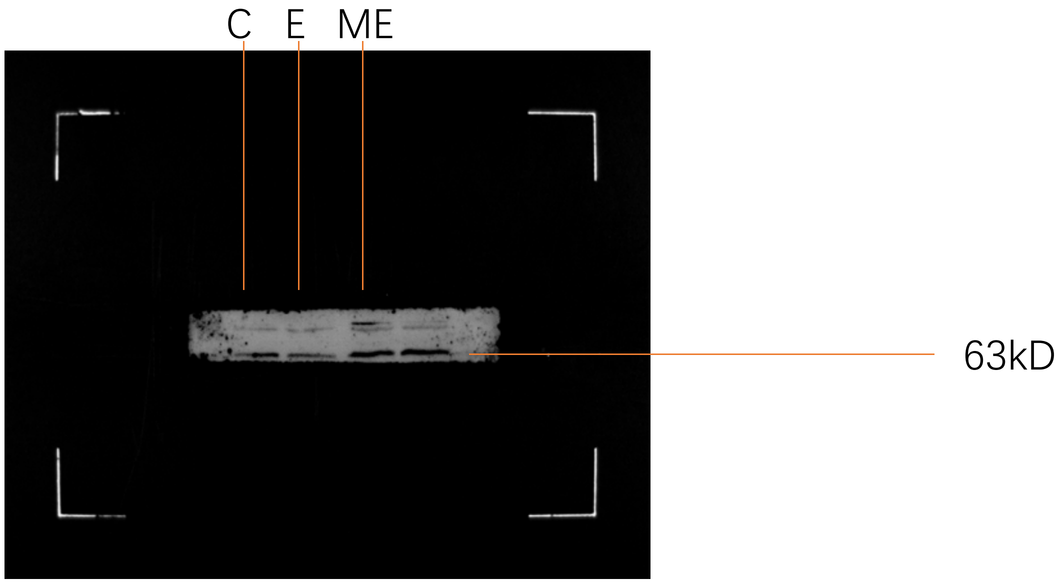

t-AMPK

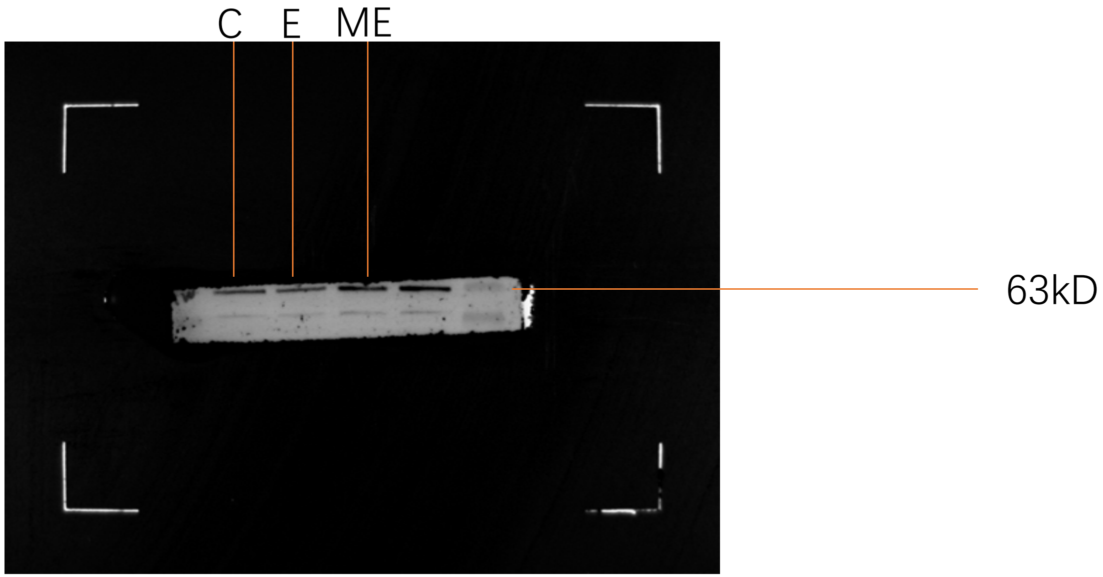

t-AMPK

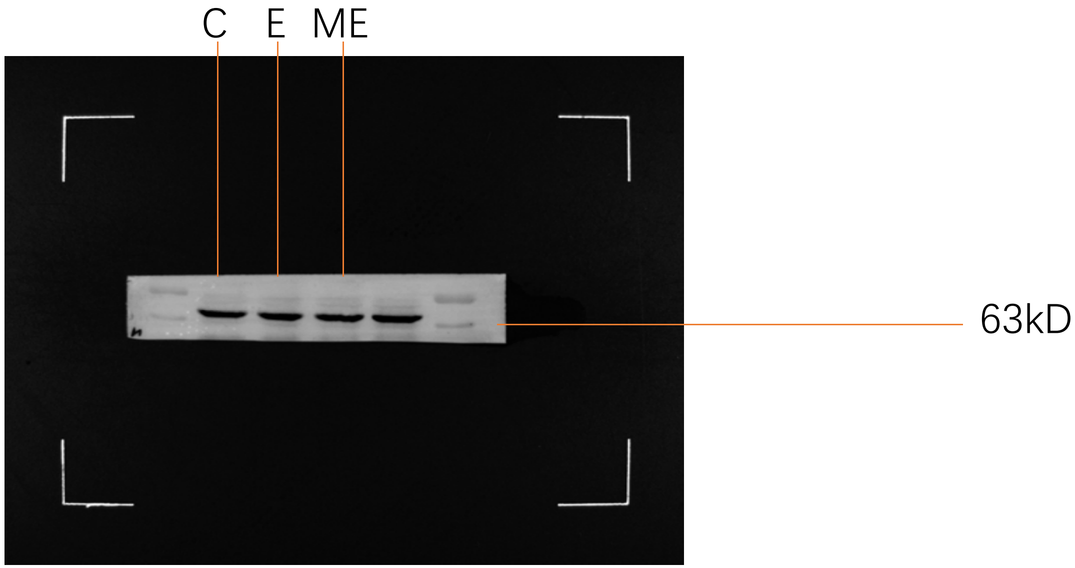

β-actin2

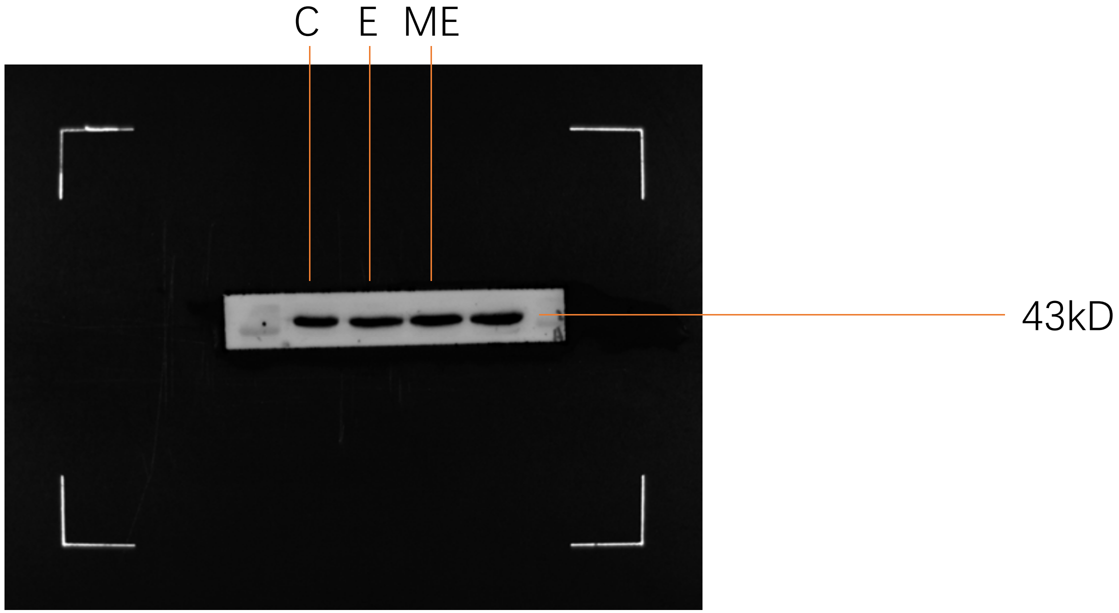

β-actin2

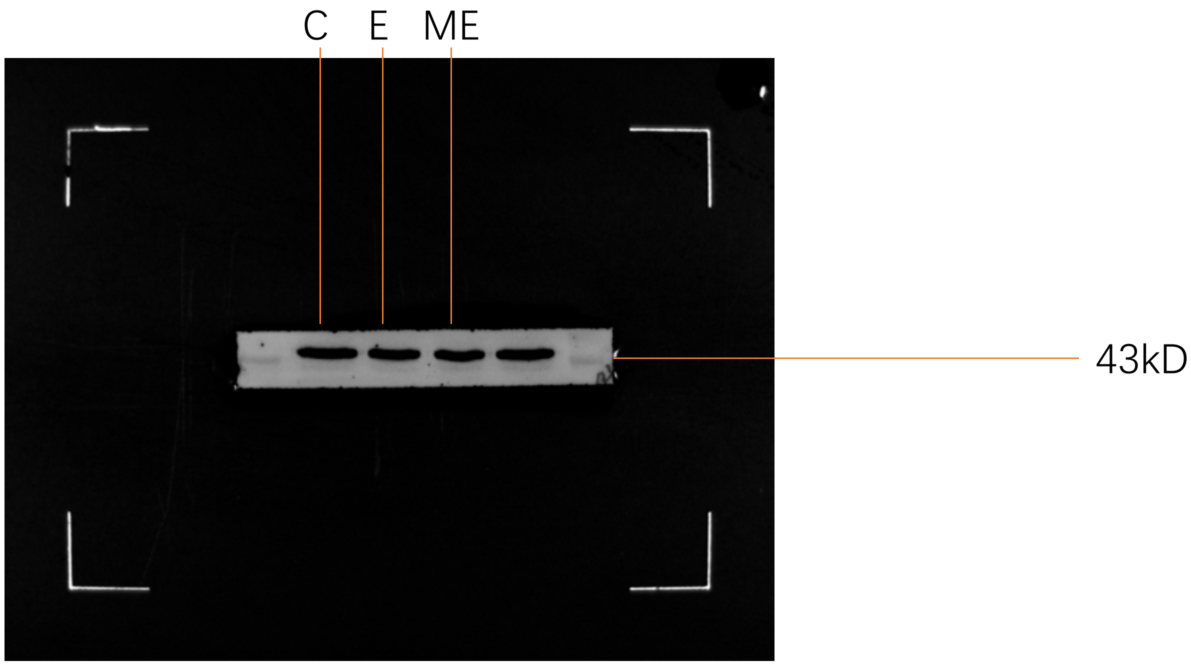

β-actin2

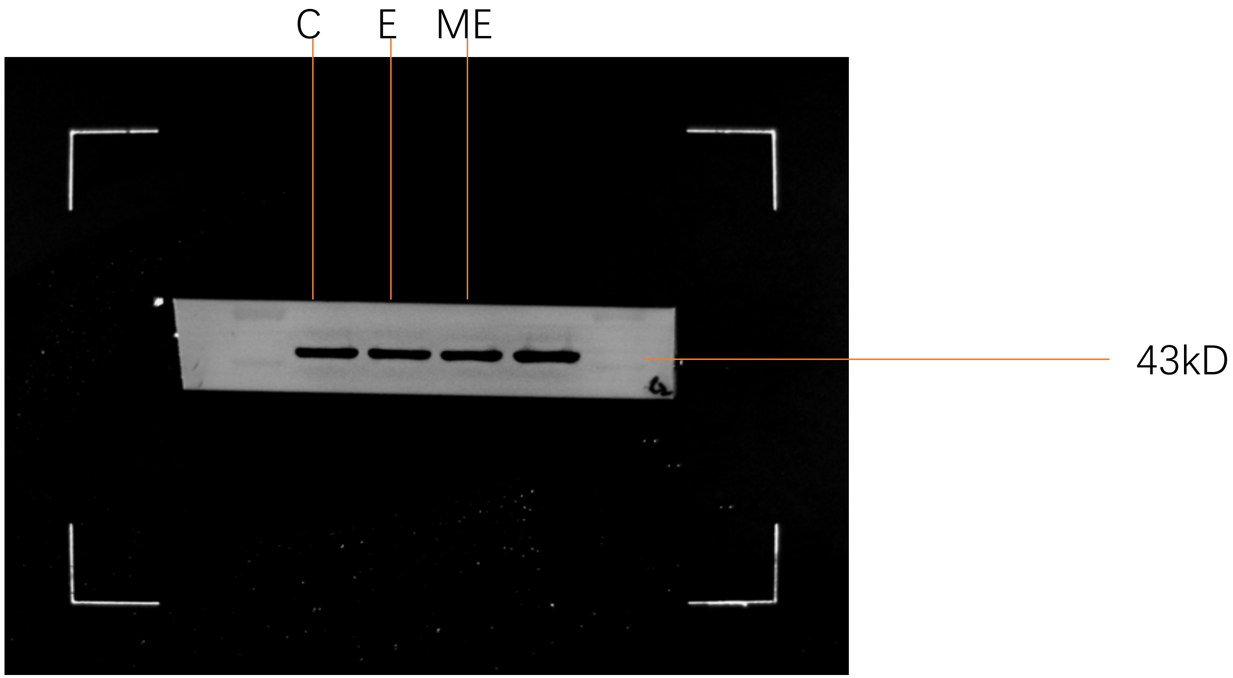

Supplement: Supplementary file 1 — Supplementary Information 1. [file 41598_2021_99568_MOESM1_ESM.pdf]
