# Supplementary figures and images for: The mitochondrial signaling peptide MOTS-c improves myocardial performance during exercise training in rats
Source: Sci Rep. 2021 Oct 11;11:20077. doi: 10.1038/s41598-021-99568-3 (PMC8505603; doi:10.1038/s41598-021-99568-3)

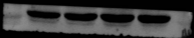

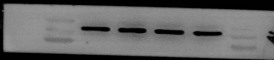

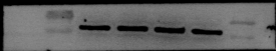

Supplement: Supplementary file 2 — Supplementary Information 2. [file 41598_2021_99568_MOESM2_ESM.pdf]

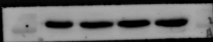

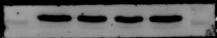

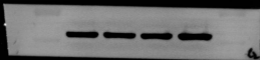

Supplement: Supplementary file 3 — Supplementary Information 3. [file 41598_2021_99568_MOESM3_ESM.pdf]

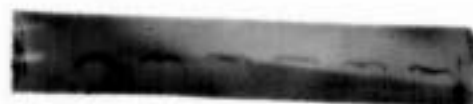

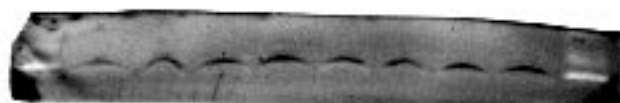

Supplement: Supplementary file 4 — Supplementary Information 4. [file 41598_2021_99568_MOESM4_ESM.pdf]

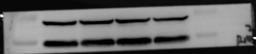

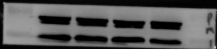

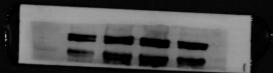

Supplement: Supplementary file 5 — Supplementary Information 5. [file 41598_2021_99568_MOESM5_ESM.pdf]

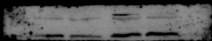

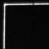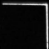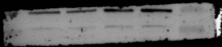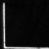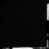

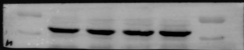

Supplement: Supplementary file 6 — Supplementary Information 6. [file 41598_2021_99568_MOESM6_ESM.pdf]
